# Navigating protected genomics data with UCSC Genome Browser in a Box

**DOI:** 10.1093/bioinformatics/btu712

**Published:** 2014-10-27

**Authors:** Maximilian Haeussler, Brian J. Raney, Angie S. Hinrichs, Hiram Clawson, Ann S. Zweig, Donna Karolchik, Jonathan Casper, Matthew L. Speir, David Haussler, W. James Kent

**Affiliations:** ^1^Center for Biomolecular Science and Engineering, School of Engineering, University of California Santa Cruz, Santa Cruz, CA 95064, USA and ^2^Howard Hughes Medical Institute, University of California Santa Cruz, Santa Cruz, CA 95064, USA

## Abstract

**Summary:** Genome Browser in a Box (GBiB) is a small virtual machine version of the popular University of California Santa Cruz (UCSC) Genome Browser that can be run on a researcher's own computer. Once GBiB is installed, a standard web browser is used to access the virtual server and add personal data files from the local hard disk. Annotation data are loaded on demand through the Internet from UCSC or can be downloaded to the local computer for faster access.

**Availability and implementation:** Software downloads and installation instructions are freely available for non-commercial use at https://genome-store.ucsc.edu/. GBiB requires the installation of open-source software VirtualBox, available for all major operating systems, and the UCSC Genome Browser, which is open source and free for non-commercial use. Commercial use of GBiB and the Genome Browser requires a license (http://genome.ucsc.edu/license/).

**Contact:** genome@soe.ucsc.edu

## 1 Introduction

Custom data display is a popular feature of modern genome browsers, allowing users to view their own private genome annotations alongside publicly available data. The University of California Santa Cruz (UCSC) Genome Browser (Karolchik *et al.*, 2014; Kent *et al.*, 2002) has supported user-generated custom annotation tracks since 2001, and recently added support for track and assembly data hubs (Nguyen *et al.*, 2014; Raney *et al.*, 2014) that allow the user to host collections of large data files on a local server and configure many details of the visualization. However, these solutions require the data files to be uploaded onto a web server or stored on a genome browser’s servers, a drawback that has become increasingly problematic as data file sizes expand and analysis involves sensitive genomic patient data. Locally installed genome browser applications like Integrative Genomics Viewer (Thorvaldsdóttir *et al.*, 2013) mitigate this problem but typically include significantly fewer genome annotations.

The UCSC Genome Browser in a Box (GBiB) circumvents these issues, offering secure local use of private data files while still providing access to the full suite of UCSC Genome Browser tools and annotations. We use an x86 virtual machine, which simulates a complete computer (‘guest’) on a normal PC (‘host’). Thanks to the hardware support offered by current CPUs, this configuration has become almost as fast as a real machine. This kind of virtualization technology is the basis of cloud computing, typically used in bioinformatics to distribute analysis tasks on-demand on machines offered by external providers (Afgan *et al.*, 2010). Virtualization tools have become very easy to install on personal computers, and therefore have been used to simplify the installation of complex bioinformatics analysis tools like Clovr (Angiuoli *et al.*, 2011) and the ENCODE project analysis pipeline (Rosenbloom *et al.*, 2013). For a website like the UCSC Genome Browser, the large amount of annotation data makes virtualization more difficult—at the time of writing, UCSC’s database includes more than 5 TB of data for the human genome alone. This exceeds the capacity of most desktop PCs and is costly to host on cloud-based computation services ($250 US/month on Amazon EBS or Microsoft Azure, as of March 2014). GBiB, by contrast, provides access to all of the same annotation data while consuming only about 20 GB of local storage.

## 2 Implementation

GBiB runs on VirtualBox, the only open-source virtualization software available on all major operating systems. It is based on standard Ubuntu Linux with an Apache web server. The installation package includes the UCSC Genome Browser CGI binaries and a minimal MySQL database needed to store user session information and selected tables. These pre-loaded tables have been selected based on their size and access frequency on the public UCSC Genome Browser website. A special tool (‘Mirror tracks’) allows a local user to save a local copy of a Genome Browser track by downloading the track data from UCSC’s servers. Any data that is not locally available is loaded transparently through the Internet from the UCSC servers.

**Fig. 1.**
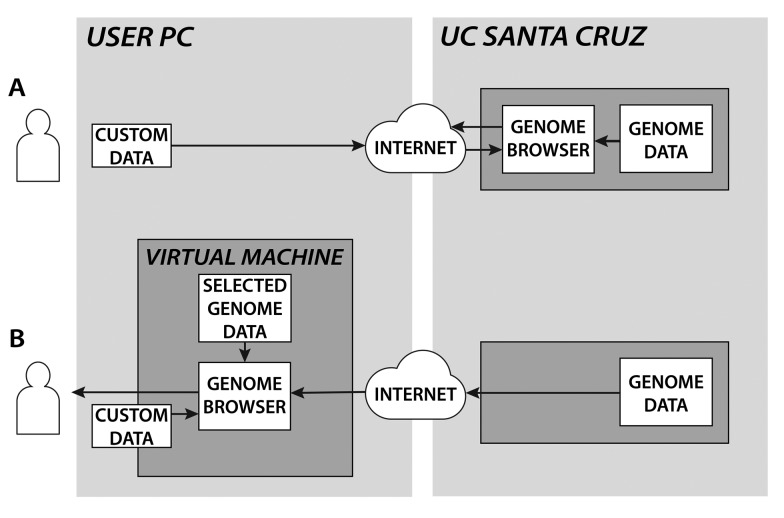
Data flow when accessing the UCSC Genome Browser website (A) versus the Genome Browser in a Box (B). In case (B) the custom track data does not leave the user’s PC and is not transmitted over the Internet

The UCSC Genome Browser stores its genome annotations in MySQL database tables or flat files, depending on the data type. Correspondingly, GBiB runs MySQL queries through the Internet when needed and transparently opens flat data files through HTTP (see Fig. 1). All file access operations have been modified to use the Genome Browser HTTP download server, using the local hard disk as a cache. Because we were unable to determine a system for caching MySQL queries on the client side in a way similar to the flat file access, we modified the Genome Browser database layer to run all queries against the local MySQL server first. Failed queries are rerun against the public UCSC MySQL server. To improve performance, database schema requests (table and field existence checks), which are relatively common in the Genome Browser, are resolved via a local copy of the database schema.

Remote data access is fast, but only when the latency to UCSC is low, as each MySQL query requires a network (TCP/IP) round trip. Because network latency depends primarily on the distance to the target computer, GBiB may be slow for users in remote locations. For example, when the latency is higher than 80 ms, the Genome Browser may require more than 5 s to draw the chromosome image. Typical latencies to UCSC are 100 ms from the US East Coast and up to 180 ms from Europe and Japan. To improve performance from these locations, GBiB includes a copy of all default annotation files and tables. It also includes a mirroring tool that simplifies local copying of annotation tracks. When a set of tracks is selected with this tool, the associated database tables and ancillary files are downloaded into the virtual machine with the high-performance UDP-based Data Transfer protocol (Gu, 2007). In addition, all local tables, data files and CGI programs are updated every few hours from UCSC’s servers.

A special problem occurs when a third-party database that supplies genome annotations for the UCSC Genome Browser does not agree to make the tables available for download on the browser website, for example, Decipher, Leiden Open-Source Variation Database, and the Human Gene Mutation Database (HGMD). We have solved this by placing the annotation track files into a password-protected portion of the UCSC website, storing the password within the Genome Browser binary file and accessing the annotations through an encrypted connection. At the time of writing, HGMD has agreed to be served to virtual machines in this way.

Although the bulk of genome annotation data is still accessed through the Internet, the big advantage of GBiB is that files can also be loaded from the host machine. VirtualBox, like many other virtualization solutions, allows the application to access directories on the local hard disk of the machine where it is running (‘shared folders’). Data in shared folders are not transmitted over the Internet by GBiB. Users can open their own protected data files and display them alongside the standard UCSC genome annotations or load a new genome as an assembly hub without fear of making their private data available to the outside world. In the unlikely event that others can monitor a user’s network traffic, they will only see which areas of the genome are accessed when remote tracks are loaded from UCSC. Tools to index or convert genome annotation formats, like BAM, VCF, BED, bigWig and twoBit (Kent *et al.,* 2010) are included in the virtual machine. Additional tools to intersect, overlap, filter or merge these files can be installed by running a simple command.

## 3 Conclusions

With Genome Browser in a Box, a researcher can run a full genome browser on almost any modern laptop. As more individual genomics data becomes available, GBiB facilitates personal and clinical genome analysis without requiring a change to the toolset many researchers are familiar with. By running a complete website on the user’s machine, our approach should be applicable to many other websites that process human genomics data, allowing the handling of protected data without transmission over the Internet.
